# Resource defense and monopolization in a marked population of ruby-throated hummingbirds (*Archilochus colubris*)

**DOI:** 10.1002/ece3.972

**Published:** 2014-02-18

**Authors:** François Rousseu, Yanick Charette, Marc Bélisle

**Affiliations:** Département de biologie, Centre d'étude de la forêt and Chaire de recherche du Canada en écologie spatiale et en écologie du paysage, Université de SherbrookeSherbrooke, Qc, J1K 2R1, Canada

**Keywords:** *Archilochus colubris*, habitat visibility, hummingbirds, intrusion rate, Quebec, resource defense, resource monopolization, space use, territoriality

## Abstract

Resource defense behavior is often explained by the spatial and temporal distribution of resources. However, factors such as competition, habitat complexity, and individual space use may also affect the capacity of individuals to defend and monopolize resources. Yet, studies frequently focus on one or two factors, overlooking the complexity found in natural settings. Here, we addressed defense and monopolization of nectar feeders in a population of free-ranging ruby-throated hummingbirds marked with passive integrated transponder (PIT tags). Our study system consisted of a 44 ha systematic grid of 45 feeders equipped with PIT tag detectors recording every visit made at feeders. We modeled the number of visits by competitors (NVC) at feeders in response to space use by a focal individual potentially defending a feeder, number of competitors, nectar sucrose concentration, and habitat visibility. Individuals who were more concentrated at certain feeders on a given day and who were more stable in their use of the grid throughout the season gained higher exclusivity in the use of those feeders on that day, especially for males competing against males. The level of spatial concentration at feeders and its negative effect on NVC was, however, highly variable among individuals, suggesting a continuum in resource defense strategies. Although the apparent capacity to defend feeders was not affected by competition or nectar sucrose concentration, the level of monopolization decreased with increasing number of competitors and higher nectar quality. Defense was enhanced by visibility near feeders, but only in forested habitats. The reverse effect of visibility in open habitats was more difficult to interpret as it was probably confounded by perch availability, from which a bird can defend its feeder. Our study is among the first to quantify the joint use of food resource by overlapping individuals unconstrained in their use of space. Our results show the importance of accounting for variation in space use among individuals as it translated into varying levels of defense and monopolization of feeders regardless of food resource distribution.

## Introduction

Resource defense behaviors, such as territoriality and temporary defense of food patches, are often explained by the spatial and temporal distribution of food resources (Brown 1964; Grant 1993). When food abundance is low, the area needed to secure enough food may be too large to efficiently expel competitors, increasing the costs of defense for an aggressive individual. Conversely, when food is extremely abundant, an aggressive individual excluding others from a food source may waste energy that could be allocated to more profitable activities, such as feeding or resting, and may expose itself to higher predation risks (Carpenter 1987; Martel 1996; Diaz-Uriarte 1999; Kim et al. 2004; LaManna and Eason 2007). Hence, resource defense should usually peak at intermediate levels of abundance as well as of spatial clumping of resources (Grant 1993; Grant and Guha 1993; Grant et al. 2002; Noël et al. 2005). This leads to variable levels of resource monopolization in a population and can thereby affect mating systems (Emlen and Oring 1977) and population dynamics (Patterson 1980; Newton 1992; Lopez-Sepulcre and Kokko 2005).

The ability of individuals to defend resources or space containing them can be influenced by many other factors (Maher and Lott 2000), most notably the level of competition (Grant 1993). High levels of competition for a resource can result in high intrusion rates in a given territory or at a given food patch, leading to high defense costs, reduced benefits associated with aggressive behaviors, and ultimately, lower monopolization (Chapman and Kramer 1996; Syarifuddin and Kramer 1996). A growing number of studies suggest that habitat structure can also play an important role in the ability of individuals to efficiently defend food resources against competitors (Eason and Stamps 1992; Basquill and Grant 1998; Hamilton and Dill 2003). Most studies found a negative relationship between habitat complexity and monopolization. In habitats where structural complexity is high, visual detection of intruders is likely more difficult because of a more obstructed field of view (Eason and Stamps 1992; Breau and Grant 2002), which leads to easier access to a defended resource for intruders. Studies that looked at the influence of habitat complexity or reduced visibility on resource defense and monopolization (Hamilton and Dill 2002, 2003) or space use (Eason and Stamps 2001) also showed a positive effect on population density (Venter et al. 2008; Dolinsek et al. 2007) and a negative effect on territory size (Breau and Grant 2002; Imre et al. 2002; Venter et al. 2008), aggression level (Basquill and Grant 1998; Corkum and Cronin 2004; Baird et al. 2006; Carfagnini et al. 2009), and in some cases, the growth rates of dominant individuals (Höjesjö et al. 2004; Hasegawa and Yamamoto 2009).

Except for certain studies on fishes (Hamilton and Dill 2003), and particularly on salmonids (Imre et al. 2002; Venter et al. 2008; Hasegawa and Yamamoto 2009), few studies examined the effect of habitat complexity in natural settings, where resources are often difficult to quantify and where individuals are unconstrained in their use of space. How individuals use space likely affects their ability to monopolize a certain area or food patch depending on the amount of time they allocate to different parts of their home ranges and to different activities, such as foraging (Hamilton and Dill 2003), feeding in other defended areas (Steingrímsson and Grant 2008), or seeking mating opportunities (Sikkel 1998; Stutchbury 1998; Sikkel and Kramer 2006). Although individual space use can be viewed as a consequence of resource distribution and competition, it can also be influenced by individual characteristics such as age or dominance status, which may in turn affect the ability of individual to defend and monopolize resources. For example, subordinate individuals can become floaters if they are not able to acquire a territory (Sergio et al. 2009), and this will likely influence their use of space. Thus, taking into account space use by individuals is essential to understand the factors influencing resource monopolization at both the individual and population level. Furthermore, despite numerous studies quantifying spatial overlap among conspecifics (Millspaugh et al. 2004; Fieberg and Kochanny 2005; Kerr and Bull 2006), its effect on resource sharing among neighboring individuals has rarely been explored, although it is what ultimately characterizes territorial behavior. Quantifying resource monopolization also allows us to characterize spatial organization as a continuum from completely undefended home ranges to totally exclusive territories (Maher and Lott 1995, 2000; Tyre et al. 2007), which better represents reality then the simple home range/territory dichotomy (Maher and Lott 1995).

In this study, we took advantage of a new technique to mark ruby-throated hummingbirds (*Archilochus colubris*) to quantify how resource monopolization and the capacity of individuals to defend food resources are influenced by competition, habitat structure, and the use of space by individuals. Our study system consists of a systematic grid of artificial feeders setup in the wild where feeders are equipped with radio-frequency identification detectors (RFID) and individuals marked with passive integrated transponders (PIT tags).

Nectarivorous birds, especially hummingbirds, have been the subject of many studies testing economic models of feeding territoriality (Carpenter et al. 1983) and investigating the links between territory size, food abundance, intrusion pressure, and investments in territorial defense (e.g., Gass et al. 1976; Norton et al. 1982; Hixon et al. 1983; Marchesseault and Ewald 1991; Eberhard and Ewald 1994; Tamm 1985; Temeles et al. 2004; Camfield 2006; Justino et al. 2012). Yet, defense and territorial behavior was often characterized in terms of territory size or investment in defense, but rarely in terms of resource monopolization. Indeed, few studies quantified the extent to which territorial hummingbirds have exclusive use of their defended area or food source, which is an essential component of territorial behavior (Pyke et al. 1996), and what factors besides food distribution and abundance affected this exclusivity. Moreover, space use by territorial hummingbirds is likely not restricted to the area defended (Powers and McKee 1994; Temeles et al. 2005), and intrusion pressure indicates that either territorial birds occasionally leave their territories or a certain proportion of the population is made of “floating” individuals. Because of the difficulty of marking and following individuals in the wild, characterization of the simultaneous use of space or of a spatially distributed resource by hummingbirds, or by any other organisms, has rarely been carried out.

Here, we addressed defense and monopolization of feeders in ruby-throated hummingbirds by modeling the number of visits made by competitors (NVC) at a given feeder in response to the number of competitors, feeder visibility, and space use by the focal individual. NVC is a measure of competitor access to food and can thus be used to quantify the level of monopolization experienced by a focal individual at a given feeder, with a higher NVC meaning a lower level of monopolization. Furthermore, the extent by which the presence of a focal individual lowers the NVC can be interpreted as a measure of its capacity or motivation to exclude competitors. Therefore, the monopolization and the capacity to defend feeders can be differentiated. For instance, if habitat preferences in hummingbirds cause a disproportionate use of feeders in open habitats compared to feeders in forest habitats, a higher NVC could be observed in open habitats strictly because of habitat preferences, resulting in lower feeder monopolization. Yet, through its defense or differential use of feeders, a focal individual could be excluding a greater number or proportion of intruders in these habitats, indicating a better defense capacity, even though the resulting level of monopolization by the individual might be lower compared to an individual defending a feeder located in a poorer habitat. We thus differentiate between monopolization and capacity to defend feeders to test four hypotheses of resource defense theory: (1) the number of competitors reduces the capacity of individuals to defend feeders and increases NVC; (2) higher visibility improves defense capacity, but also causes a higher NVC linked to habitat preferences; (3) higher spatial concentration and stability of focal individuals in their use of feeders lead to a lower NVC. We also manipulated sugar concentration in feeders to assess the influence of resource quality on monopolization and defense of feeders. Specifically, we tested the hypothesis that (4) high food quality leads to greater defense of feeders, but results in lower monopolization because of higher competition for high-quality feeders.

## Methods

### Study system

**Figure 1:**
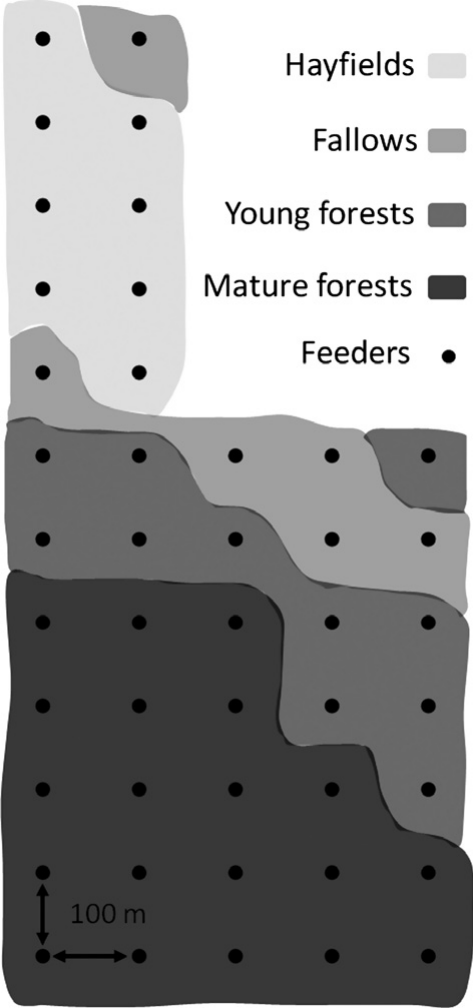
Schematic view of habitats within the feeder grid providing a nectar sucrose solution for Ruby-throated Hummingbirds in Cleveland County, Quebec (Canada), 2007–2009.

We conducted field work during the breeding seasons (20 May–30 August) of 2007–2009 in Cleveland County, Quebec, Canada (45°, 40′N; 72°, 05′W). Our study system consisted of a grid of 45 feeders distributed systematically over 44 ha (Fig. 1). Feeders were spaced 100 m apart and were set up in two rows of 12 feeders followed by three rows of seven feeders. The grid covered a gradient of vegetation cover, going from hayfields and fallows to mature deciduous and mixed forests (eight feeders in hayfields, six in fallows, and 31 in heterogeneous forested areas). The grid thus provided a uniform distribution of feeders, which standardized food distribution across the grid and therefore eliminated the effect of food distribution on the ability of individuals to monopolize certain feeders. Differential use of feeders was then ultimately determined by surrounding habitat features and interactions between individuals.

Feeders (Yule Hide, model HB81, capacity: 455 mL) were red and included a single opening mimicking a yellow flower. They were installed on metal poles at a height of 1.3–1.7 m and covered by an aluminum plate to prevent evaporation and excessive heating from direct sunlight. We changed feeders once a week by sterilized ones filled with a solution of 20% (W/V of solvent) of sucrose, a concentration similar to the nectar of natural flowers (Baker 1975; Bolten et al. 1979; Roberts 1996). Each feeder was equipped with an antenna hooked to a radio-frequency identification reader (Trovan Electronic Identification System, model LID650, model ANT 614 OEM; 5 × 8 cm, East Yorkshire, U.K.). The antenna consisted in a rectangular-shaped copper wire attached to the only perching site of the feeder. The antenna was set up vertically on the perch so that birds were forced to perch in the antenna while drinking, which enabled detection. The readers were programmed to scan for passive integrated transponders (PIT tags) every second. Hence, we recorded every second (date and time of day) that a tagged individual spent in the antenna along with its tag ID. We transformed those readings in visits such that every visit by an individual was characterized by a start time and duration. Because hummingbirds often perform small, back-and-forth movements (≪ 1 m) while foraging at feeders (or flowers) and because detectors occasionally skipped some readings, we considered that two consecutive readings by the same individual at a given feeder and <21 sec apart were part of the same visit. Detectors were active on a 24 h/day schedule.

The ruby-throated hummingbird is known to aggressively defend natural and artificial food sources, especially in the case of adult males (Robinson et al. 1996). Males presumably defend territories centered on food sources that may also play a role in mate acquisition (Pitelka 1942). Like in most hummingbird species, males provide no parental care and their role in reproduction is restricted to mating (Robinson et al. 1996), implying that space use is likely to differ substantially between sexes. High variability in male reproductive success (Mulvihill et al. 1992) could also imply varying levels of aggression and efficiency at defending food sources and multiple spatial strategies among males.

### Capture and marking

We captured hummingbirds near or at feeders using mist nets (36 mm or 28 mm mesh) or Hall traps (Russell and Russell 2001). Capture was usually carried out between 06:00 h and 13:00 h throughout the study period. Although capture efforts were oriented toward feeders where unmarked individuals were seen during standardized focal observations, we ensured that all feeders were subjected to a minimum capture effort within a 10-day period. We also increased capture efforts when unmarked individuals were seen and when hummingbird activity on the grid was high. We fitted individuals with an aluminum leg band, and we glued the PIT tag (Trovan Electronic Identification System, model ID100A; weight: 0.09 g; size: 2.12 × 11.50 mm, East Yorkshire, U.K.) on the back feathers in the interscapular region. The leg band, the PIT tag, and the glue represented 5% or less than the hummingbird's body mass, which is acceptable according to established standards (Kenward 1987).

### Space use and competitors

We quantified space use by focal individuals using indices of spatial concentration and stability. Spatial concentration was defined as the ratio between the number of visits by an individual to a given feeder on a given day and its total number of visits on the grid for the same day. Stability was defined as the linear correlation between spatial concentration and seasonal spatial concentration. Seasonal spatial concentration was the same measure as spatial concentration but was calculated for the entire period during which an individual was followed. The correlation between these measures therefore represents the level to which the daily use patterns of the grid mimicked the seasonal pattern, which can be seen as an index of spatial stability or fidelity in the usage of the grid by an individual. It varies from 0 to 1, with 1 representing total stability. Because spatial stability requires at least 3 days to be evaluated, only individuals that were followed for more than 2 days were considered as focal individuals (mean ± SD of number of days followed: males = 38.3 ± 22.2, females = 21.4 ± 16.5), although all adults were included in the calculation of the number of competitors (daily number of individuals detected at the feeder) and NVC.

Competitors should not be restricted in their access to a feeder unless it is defended. We thus expected that spatial concentration would have no effect on NVC if an individual did not defend feeders. On the other hand, we expected a negative effect of spatial concentration on NVC for an individual that aggressively defends feeders. Assuming that spatial concentration reflects the importance of a feeder to an individual, the more a defending individual is concentrated at a given feeder, the greater should be its negative impact on NVC at that feeder. The effect of spatial concentration on NVC thus reflects, at least partially, the ability and/or the willingness to defend of an individual, and the strength of this effect represents the degree or the effectiveness of its defense. Therefore, looking at the effect of different variables on the ability of individuals to efficiently defend feeders required that we include interactions between spatial concentration and variables thought to affect this ability in our models. For instance, if the ability of individuals to defend feeders is reduced by the number of competitors, the negative effect of spatial concentration on NVC should be weaker when the number of competitors is high. We did not categorize individuals as either territorial or nonterritorial because of the difficulty of identifying and tracking individuals in the field by sight, and more importantly, because we feel the dichotomy between these two extremes is too rigid. Indeed, it is well known that hummingbirds can adjust their intensity of defense in response to the quality of their territories or the quality of their defended resources (e.g., Ewald and Bransfield 1987, Camfield 2006; Justino et al. 2012). Consequently, we assumed that the level of territoriality and resource defense would be reflected in the strength of the reduction in NVC with an increase in spatial concentration.

### Visibility

We assessed visibility around feeders using two habitat variables. First, each feeder was categorized as being in an opening or not. An opening was defined as a >50 m^2^ gap in the canopy. Fourteen of the 31 feeders located in forest habitat fell in the former category along with all of the 14 feeders situated in hayfields and fallows. Second, lateral visibility between 1 and 2 m in height was measured around each feeder at eye's height (∼1.5 m). It was defined as the distance (m) at which ≥90% of a banner (width = 30 cm) located at the feeder was visible by an approaching observer. This measure was evaluated by the same observer at all feeders and averaged over the four cardinal directions. We assumed that feeders located in openings were in high-visibility environments, independently of lateral visibility, while lateral visibility in closed environments was a better indicator of general visibility for a hummingbird at the feeder. This assumption was made because hummingbirds usually perch high in open habitats and thereby have an overview of feeders unobstructed by the low shrubby vegetation that may occur in these habitats. In order to model the influence of visibility on the relationship between spatial concentration and NVC, we thus had to include a three-way interaction among openness, lateral visibility, and spatial concentration. Indeed, we expected the negative effect of spatial concentration on NVC to be independent of lateral visibility in open habitats, but to increase with lateral visibility in closed habitats.

### Food quality

We manipulated feeder quality in 2009 by increasing the sucrose concentration of some feeders from 20 to 35%. Previous studies showed that hummingbirds preferred nectar of high sucrose concentration and that this preference peaked somewhere between 40 and 65% across studies (e.g., Tamm and Gass 1986; Roberts 1996; Blem et al. 2000). Although our measurement units may differ from other studies, our high-concentration treatment is close to the range of preferred concentrations (Bolten et al. 1979). Moreover, Camfield (2006) found higher intrusion rates by rufous (*Selasphorous rufus*) and broad-tailed hummingbirds (*S. platycercus*) at feeders filled with a 30% (W/V) sucrose nectar compared to feeders containing a 20% sucrose nectar. We are therefore confident that a 35% solution represents higher quality nectar compared to a 20% solution. Our manipulation was divided into three blocks of 3 weeks each, lasting from 10 June to 11 August. Each week, we randomly assigned the high concentration to 15 of the 45 feeders with the constraint that every feeder had to be of high concentration exactly once throughout a block, ensuring a complete coverage of feeders within 3 weeks. As the low-concentration treatment corresponded to the standard sucrose concentration found in the first 2 years, we ran analyses using observations from all 3 years (i.e., 2007–2009) while assigning the low-concentration treatment to all feeders of 2007 and 2008; analyses performed exclusively on the 2009 data gave very similar effect sizes, yet with larger standard errors as expected from the lower number of observations.

### Sex

Because of the numerous interactions that could arise from the inclusion of sex as an explanatory variable, we chose to restrict our analyses to the four possible sex combinations of focal individuals and competitors, that is, the effect of focal males on male or female competitors and the effect of focal females on male or female competitors. This approach allowed us to determine the extent of intra-and intersexual territoriality, while reducing model complexity. As the level of defense in other combinations seemed much lower, we decided to focus on male versus males interactions in the main part of this study as the level of defense in other cases may not be strong enough to study the influence of factors other than spatial concentration on food resource defense and monopolization. Results regarding combinations other than the males versus males competitors are presented in Appendix 1.

### Control variables

The total daily number of visits by the focal individual was included to account for the fact that a spatial concentration of 90% at a given feeder was unlikely to have the same impact on NVC if the individual made 10 visits on the grid compared to 100 visits. To control for variable meteorological conditions and availability of natural food sources that may affect the level of feeder use, we derived an index corresponding to the mean daily number of visits across hummingbirds that used the grid. For example, if temperatures are low, feeder use by competitors will likely increase in response to higher energetic demands. We thus used this index as an estimate of the relative reliance on feeders across days which will likely be reflected in the NVC. To control for the fact that certain feeders may be more attractive to hummingbirds, we ranked feeders according to the number of different individuals detected at the feeder at least once throughout the season. Feeders were ranked in ascending order with the feeder with the highest number of individuals detected having the lowest value, with individuals restricted to the sex of competitors. Ranks were consistent across the 3 years of study with a high interannual correlation (*r* = 0.89–0.95, *n* = 45), suggesting that feeder attraction was maintained through time. We restricted analyses to adults as juveniles were detected only at the beginning of August and most stayed on the grid only for a few days. We were not able to consider the age of individuals, which might affect territorial behavior or dominance (Ewald 1985; Carpenter et al. 1993), because aging adults in ruby-throated hummingbirds is only possible through recapture of individuals initially captured as juveniles, which seldom occurred in our study system.

### Statistical analyses

To determine whether individuals were more concentrated at a certain feeder than what would be expected from a random use, we determined the spatial concentration for every individual at their most visited feeder on each day and calculated the mean daily spatial concentration with every individual-day combination. We then compared this observed value to 100 mean daily spatial concentration values generated by randomly assigning the visits made by an individual to every feeder it visited on a given day for every individual-day combination. A similar procedure was used to determine whether the pattern of use of a given feeder on a given day by different individuals is indicative of a single individual being dominant at that feeder in terms of number of visits. We randomly assigned every visit made at a feeder to individuals visiting it on that day. We then calculated with every feeder-day combination the mean number of visits performed by the individual that made the most visits to the feeder. This procedure was repeated 100 times, and the observed mean number of visits was compared against the random values.

We used linear mixed models to quantify the influence of explanatory variables on the number of visits made by competitors (NVC) in relation to a focal individual, with feeder ID and focal individual ID as random effects. Competitors were defined in relation to a focal individual at a given feeder. Hence, every individual that visited a given feeder on a given day was in turn considered as a focal individual potentially defending a feeder while other visitors were considered as its competitors in the calculation of NVC. Although unmarked individuals could occasionally be seen at feeders, the correlation between the yearly mean number of visits at feeders detected through PIT tags and the yearly mean number of visits and pursuits detected during standardized bi-weekly focal observations is 0.68, indicating that there is a good relation between hummingbird activity at feeders and what is detected through PIT tags. We also expected individuals to vary in their will and ability to defend feeders and thereby allowed the slope characterizing the effect of spatial concentration on the reduction in NVC to vary as a random parameter across focal individuals. We log-transformed NVC to meet assumptions of normality and homoscedasticity. Cases where only one individual was detected at a feeder on a given day were excluded as there is no variation in NVC in such cases and the lack of a competitor more likely represents a feeder less attractive to hummingbirds than a feeder that is perfectly defended, assuming that a perfect exclusion of competitors is unlikely. The time spent at feeders by competitors was also used as a response variable and results were highly similar. Only analyses based on NVC are thus presented here. We selected models and performed multimodel inference based on the Akaike Information Criterion corrected for small sample sizes (AIC_*c*_) following Burnham and Anderson (2002) and Vaida and Blanchard (2005). All models included year, grid usage, number of competitors, feeder rank, and nectar sucrose concentration as these variables were mostly used as controls (model 1; Table 1). Model (2) contained variables related to the habitat surrounding feeders while model (3) also included variables related to the characteristics of focal individuals that could influence NVC. Following models were built by leaving out single variables or group of variables and/or their interactions with spatial concentration, which represent their relation or their effect on feeder defense by focal individuals. Models (4) and (5) assess whether a greater daily use of the grid or spatial stability across the season by the focal individual is associated with a stronger defense of feeders. Models (6) and (8) evaluate whether number of competitors or habitat structure influences the capacity to defend feeders. Models (7) and (8) differentiate between the effect of habitat structure on the general use of feeders or on the capacity of the focal individual to exclude competitors. Model (9) assesses whether nectar sucrose concentration of feeders is associated with a greater defense. Finally, model (10) contained all explanatory variables and interactions of interest. AIC_*c*_ values were computed based on the models' maximum likelihood and model averaging performed on coefficients obtained by restricted maximum likelihood. Analyses were conducted in R 2.10.1 (R Development Core Team 2009) using the lmer function from the lme4 package (version 0.999375-32).

**Table 1:** Model selection and explanatory variables composing the 10 models put in competition by AICc for modeling the number of visits by male ruby-throated hummingbird competitors in Cleveland County, Quebec (Canada), 2007–2009. Variables included and omitted from a model are indicated by a cross and a circle, respectively. Akaike weights (*w*_*i*_) represent the probability that a particular model best describes the data. The response variable was log-transformed and modeled with linear mixed-effect models with feeder ID and the intercept and slope of spatial concentration for focal individual ID as random effects.

Variables	1	2	3	4	5	6	7	8	9	10
Year	x	x	x	x	x	x	x	x	x	x
Grid usage	x	x	x	x	x	x	x	x	x	x
nb of competitors	x	x	x	x	x	x	x	x	x	x
Feeder rank	x	x	x	x	x	x	x	x	x	x
Nectar sucrose concentration	x	x	x	x	x	x	x	x	x	x
Spatial concentration	o	o	x	x	x	x	x	x	x	x
Daily nb of visits	o	o	x	o	x	x	x	x	x	x
Spatial stability	o	o	x	x	o	x	x	x	x	x
Openness	o	x	x	x	x	x	o	x	x	x
Lateral visibility	o	x	x	x	x	x	o	x	x	x
Spatial concentration:daily nb of visits	o	o	o	o	x	x	x	x	x	x
Spatial concentration:nb of competitors	o	o	o	x	x	o	x	x	x	x
Spatial concentration:spatial stability	o	o	o	x	o	x	x	x	x	x
Spatial concentration:openness:lateral visibility	o	o	o	x	x	x	o	o	x	x
Spatial concentration:feeder Concentration	o	o	o	o	o	o	o	o	o	x
ΔAICc	911.79	901.67	77.68	34.47	2.01	0.00	39.58	26.87	0.24	2.15
*w*_*i*_	0.00	0.00	0.00	0.00	0.14	0.39	0.00	0.00	0.34	0.13

## Results

**Figure 2:**
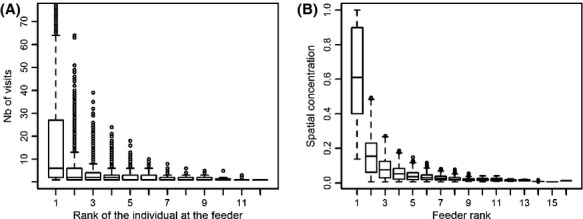
Boxplots showing number of visits and spatial concentration of adult Ruby-throated Hummingbirds in Cleveland County, Quebec (Canada), 2007–2009. (A) For a given feeder on a given day, every individual has been ranked according to its number of visits at the feeder, with rank 1 being the individual with the most visits to the feeder. This figure shows how the number of visits at feeders decreased with the rank of individuals. For individuals of rank one, 427 points are over 75 visits (max = 188 visits) and are not shown on the figure. (B) For a given individual on a given day, every feeder has been ranked according to the number of visits made by the individual, with rank 1 being the most visited feeder. This figure shows the decrease in spatial concentration of individuals in relation to feeder rank. Both graphics are for the male versus males combination only.

Over the 3 breeding seasons, we followed 88 focal males, representing 3326 bird-days and 15 037 bird-feeder-days. Individuals that made the most visits to a given feeder on a given day made a disproportionately higher number of visits than other individuals (Fig. 2A) (mean number of visits by individual of rank 1 = 19.29; random values (mean ± SD) = 7.88 ± 0.03; *P* < 0.01). Moreover, most if not all individuals made a disproportionately higher proportion of their visits at a single feeder (Fig. 2B) (mean spatial concentration at feeder of rank 1 = 0.631; random values (mean ± SD) = 0.378 ± 0.002; *P* < 0.01). In spite of considerable variation among individuals, those results suggest that feeders are used more or less exclusively, that individuals are moderately to highly concentrated in space, and that in most cases, every individual can be linked to a “primary” feeder.

**Figure 3:**
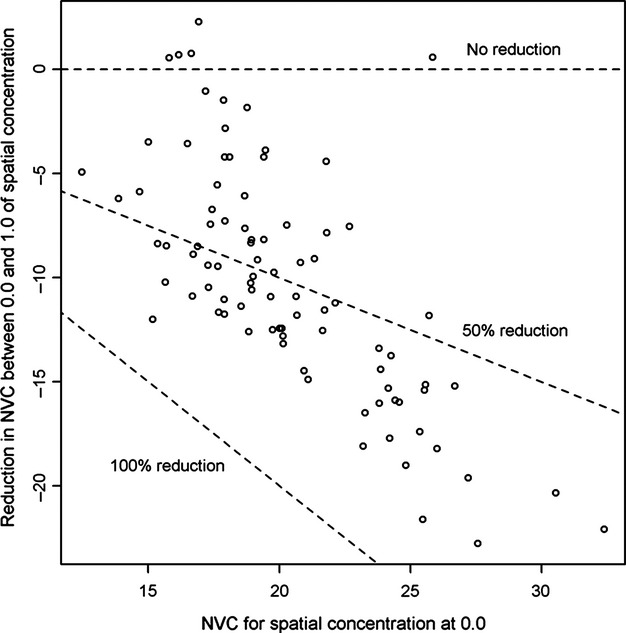
Predicted reductions in the number of visits made by adult male ruby-throated hummingbird competitors (NVC) when focal males' spatial concentration goes from 0.0 to 1.0 in Cleveland County, Quebec (Canada), 2007–2009. The *X* axis represents NVC when spatial concentration is 0, that is, when the focal individual is presumably absent from the feeder and when competitors have a free access to the feeder relative to the focal individual. Values were obtained using the model-averaged best linear unbiased predictors (BLUPs) of the linear mixed models listed in Table 1 as individuals were treated as random effects for the intercept and the slope characterizing the effect of spatial concentration. Other numeric variables were fixed to their mean value, and factors were fixed to their reference level.

Models that did not consider the spatial concentration of individuals, either as a main effect or in interactions, were the least supported by the data.(Table 1, model 1–2), which clearly implies that resource defense plays an important role in reducing the NVC in male–male interactions. Indeed, when spatial concentration varied from 0.0 to 1.0, NVC was reduced by 53%. Model selection also indicated that the influence of spatial concentration on NVC varied among individuals. A comparison between full models with and without a random effect that allowed the slope of spatial concentration to vary among individuals showed a clear differential support for treating this parameter as random (ΔAIC_c_ = 86.2). Variability among focal males was particularly high, with some individuals showing no, or even a positive effect of spatial concentration on NVC, while others showed a strong reduction in NVC with increasing spatial concentration (Fig. 3). Thus, the level of defense by males seems to be characterized by a continuum from no apparent defense to a strong defense level of feeders, although most individuals showed a moderate level of defense.

**Figure 4:**
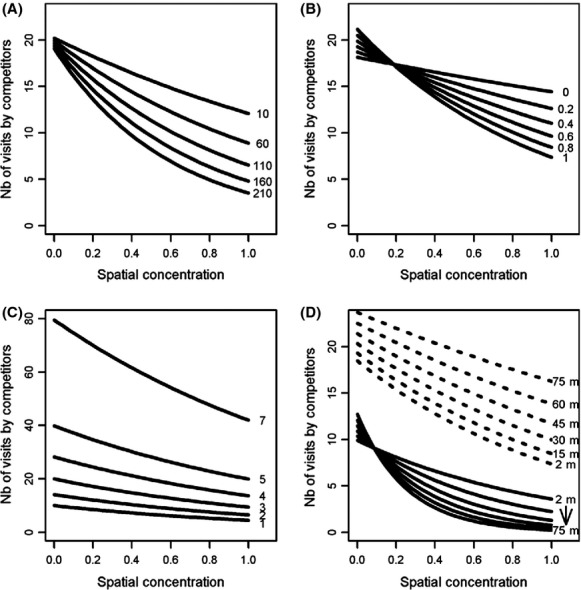
Model-averaged predictions for the number of visits made by male ruby-throated hummingbird competitors (NVC) in Cleveland County, Quebec (Canada), 2007–2009, in relation to the spatial concentration of the focal male and (A) the total number of visits by the focal individual, (B) the spatial stability of the focal individual, (C) the number of competitors, and (D) the lateral visibility (meters) and habitat openness (open; gray lines, closed; black lines). Predictions are derived from a linear mixed model with feeder ID and the intercept and slope of spatial concentration for focal individual ID as random effects. The response variable was log-transformed. Other numeric variables were fixed to their mean value, and factors were fixed to their reference level.

**Figure 5:**
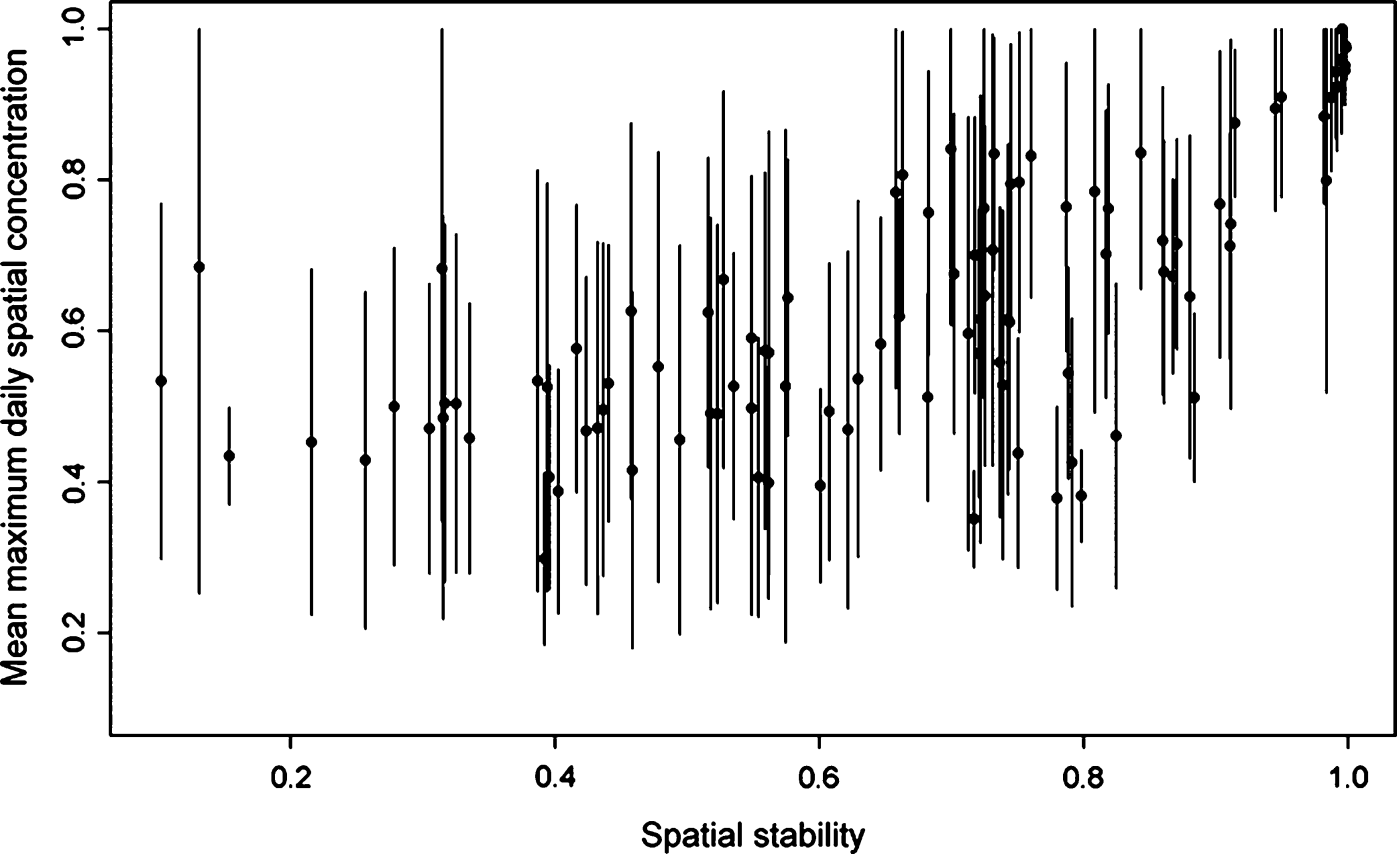
Relation between the mean maximum daily spatial concentration and spatial stability of adult male Ruby-throated Hummingbirds in Cleveland County, Quebec (Canada), 2007–2009. The mean maximum daily spatial concentration represents the average spatial concentration of an individual at its most visited feeder on a given day. For cases in which an individual was followed for more than one season, the mean was calculated across all season without distinction of the year. Lines correspond to the standard deviation for every individual. Although there seems to be a positive association between spatial stability and the mean maximum daily spatial concentration, individuals stable in space are not necessarily concentrated.

The negative effect of spatial concentration on NVC was more pronounced when the daily total number of visits made to the grid by the focal individual was high, that is, when it made a greater use of the grid (Table 2, Fig. 4A). The negative effect of spatial concentration also became stronger with increasing spatial stability, indicating that individuals concentrated and stable in space gained higher exclusivity in the use of feeders (Table 2, Fig. 4B). Although spatial stability and spatial concentration showed a weak association, there was a high variation in the daily spatial concentration of individuals (Fig. 5). NVC increased with the number of competitors, but this increase did not depend on the level of spatial concentration, which suggests that an individual's capacity to defend feeders was not affected by the number of competitors (Table 2, Fig. 4C). Habitat structure also influenced the capacity of individuals to defend and monopolize feeders. When feeders were in open habitat, contrary to our prediction, the negative effect of spatial concentration slightly decreased with lateral visibility (Table 2, Fig. 4D). In closed habitat, however, the interaction was reversed and the negative effect of spatial concentration increased with lateral visibility as we predicted. Although these results suggest that capacity to defend feeders is influenced by visibility, a high capacity to exclude competitors from feeders does not necessarily imply a higher degree of monopolization. Indeed, NVC was much higher in open habitats than in closed ones, which may indicate a disproportionate use of feeders in open areas. To achieve the same degree of monopolization or NVC, an individual defending a feeder in the open would thus have to exclude more intruders than an individual defending a feeder in closed habitat, where intrusion rates are probably lower. Finally, although the daily number of visits by male competitors was higher at 35% sucrose nectar feeders than at 20% feeders (Appendix 2), the negative effect of spatial concentration on NVC was not affected by nectar sucrose concentration (Table 2).

**Table 2:** Model-averaged coefficients (coef), unconditional standard errors (SE) and 95% confidence intervals (lower CI and upper CI) for the explanatory variables used for modeling the number of visits made by adult male ruby-throated hummingbirds competitors (NVC) at feeders potentially defended by a male in Cleveland County, Quebec (Canada), 2007–2009. The response variable was log-transformed and modeled using linear mixed models with feeder ID and the intercept and slope of spatial concentration for focal individual ID as random effects. Results are based on 88 focal individuals monitored for a total of 3326 bird-days and 15 037 bird-feeder-days.

Variables	Coef	SE	Lower CI	Upper CI
Year 2008	−0.09017	0.03778	−0.16423	−0.01612
Year 2009	−0.17854	0.04631	−0.26931	−0.08777
Grid usage	0.01735	0.00090	0.01560	0.01911
nb of competitors	0.34542	0.00571	0.33423	0.35660
Feeder rank for males	0.01531	0.00328	0.00889	0.02173
Nectar sucrose concentration (high)	0.51430	0.03940	0.43708	0.59152
Spatial concentration	−0.20918	0.21631	−0.63314	0.21479
Daily nb of visits	−0.00028	0.00049	−0.00123	0.00067
Spatial stability	0.15381	0.11210	−0.06591	0.37353
Openness (open)	0.62874	0.20028	0.23620	1.02129
Lateral visibility	0.00347	0.00395	−0.00427	0.01122
Spatial concentration:daily nb of visits	−0.00587	0.00120	−0.00822	−0.00352
nb of competitors:spatial concentration	0.02693	0.01979	−0.01186	0.06572
Spatial concentration:spatial stability	−0.81189	0.31734	−1.43387	−0.18991
Spatial concentration:feeder concentration (high)	0.03809	0.13691	−0.23026	0.30644
Spatial concentration:openness (close):lateral visibility	−0.04051	0.01601	−0.07189	−0.00914
Spatial concentration:openness (open):lateral visibility	0.00744	0.00170	0.00411	0.01078

## Discussion

To our knowledge, our study is the first to quantify the defense and monopolization of food resources within a marked population of wild animals that range freely over a spatial scale that can encompass several territories. In spite of the fact that numerous studies quantified the degree of spatial overlap among individual home ranges or territories (Millspaugh et al. 2004; Fieberg and Kochanny 2005), few have linked this overlap with the joint use of food resources even though food partitioning is often one of the main consequences of territorial behavior. By monitoring the access of individuals to available food sources, we were able to show that the relative spatial concentration and the stability in space use patterns of breeding male ruby-throated hummingbirds at specific sources of nectar increased their monopolization of those sources toward other males. Those findings, along with the fact that we documented that food resource defense dynamics depends on the sex of defenders and competitors (Appendix 1) and that individuals vary strongly in their level of food resource defense and monopolization, clearly support Maher and Lott's (2000) claim that one should address territoriality within a multivariate context.

The sharp decrease in the number of visits to a given feeder on a given day between the individual that made the most visits and other individuals that visited the feeder suggests that feeders were monopolized to some degree (Fig. 2A). However, although many individuals were able to clearly dominate in terms of visits to a given feeder, the level of monopolization of feeders was highly variable. Despite studies showing that hummingbirds will aggressively defend food sources providing *ad libitum* artificial nectar (e.g., Camfield 2006), the incentive for defense may have been relaxed in our system. Indeed, reduced defense when food is overabundant or unlimited has been shown in hummingbirds as well as in other taxa (e.g., Toobaie and Grant 2013), with lower investments in fights for the most productive feeders in black-chinned hummingbirds (*Archilochus alexandri*) (Ewald 1985) and a smaller percentage of interspecific competitors being chased by blue-throated hummingbirds (*Lampornis clemenciae*) when food was very abundant (Powers and McKee 1994). It remains that conspecifics were still being chased at a high rate in the latter study, which suggests that food defense in this species has other functions than energy acquisition, such as acquiring mates. Our observed decrease in the number of visits with the rank of the individual at the feeder could also be due to avoidance of conspecifics instead of active defense in which case our conclusions about the effects of explanatory variables on feeder defense could be erroneous. However, standardized 10-min focal observations we conducted weekly at feeders showed that pursuits and aggressive interactions (involving individuals of the same sex or not) were common (16.9% of 2459 feeder visits resulted in a pursuit, F. Rousseu, Y. Charette and M. Bélisle, unpubl. data), indicating that even if conspecific avoidance was present, there was an active defense of feeders as well.

### Individual variation and space use

The variability among male ruby-throated hummingbirds in their spatial concentration and the negative effect of spatial concentration on the number of visits by male competitors show that space use must be taken into account to assess how individuals overlap in their use of resources and/or space. Furthermore, the high individual variation in spatial concentration and its effect and the interaction between spatial concentration and stability suggest that males adopt different strategies with respect to the defense of nectar sources. Indeed, high spatial concentration and high spatial stability in the use of feeders might be indicative of individuals adopting a more territorial strategy while low concentration and low stability could be linked to more sporadic use of feeders or to floating individuals unable to successfully defend a specific area (Lenda et al. 2012), although both ends might reflect a continuum in our system rather than two opposing strategies. As territory quality has been shown to influence male mating success in hummingbirds (Temeles and Kress 2010), the high variation in mating success reported in male ruby-throated hummingbirds (Mulvihill et al. 1992) may thus result from more dominant individuals being able to defend better areas. The variation in defensive behaviors found on our grid likely implies variation in fighting abilities among males which could then translate to varying level of mating success. Because aging adult ruby-throated hummingbirds based on plumage is impossible, we were not able to relate age to the space use and defense strategies adopted by individuals.

As feeder use patterns were studied without consideration of the daily temporal pattern of visits, it is possible that we have underestimated the degree of exclusivity in the use of feeders by breeding male ruby-throated hummingbirds. Indeed, the temporal nature of data in studies measuring the spatial overlap among defended areas is often neglected although it can provide significant insights regarding the joint use of space or resources by individuals (Minta 1992; Kernohan et al. 2001). By visiting or temporally defending several feeders, male ruby-throated hummingbirds may cover larger areas and thereby gain greater access to females, although the relative importance of male and female mobility and territorial behavior for mating success in ruby-throated hummingbirds remains unknown.

### Competition

The rapid increase in the number of visits by competitors with the number of competitors we have detected at feeders indicates lower resource monopolization with increased competition as observed in many studies, independently of taxa (e.g., Chapman and Kramer 1996; Syarifuddin and Kramer 1996). On the other hand, the lack of a significant interaction between spatial concentration and the number of competitors suggests that the effectiveness of defense was not overly affected by competition. This result may, however, be due to competition levels that were not sufficient for some individuals to cease defending feeders. Such a situation may be typical of what occurs on the breeding grounds compared to migratory stopovers where several hummingbirds can often be observed feeding simultaneously at one feeder with barely any chasing among individuals (Y. Charette, pers. obs.).

### Habitat structure and visibility

As predicted, the negative effect of spatial concentration was stronger when lateral visibility was high in closed habitats. Yet, the inverse relationship we observed in open habitats was unexpected if we assume that overall visibility was likely better in these environments. One variable that may have confounded our results is the availability of perches around feeders. Indeed, prominent perches can be important in hummingbirds for detecting and displaying to females (Armstrong 1987) and likely provide standpoints that facilitate competitor detection in territorial organisms (Switzer and Walters 1999). As feeders with high lateral visibility in open habitats are mostly found in hayfields, where perches are often located far from feeders in hedgerows bordering the fields, the reduced effect of spatial concentration with high lateral visibility in open habitats may therefore result from a low availability of good perches. Given the difficulty of quantifying perch availability, one may have to design an experiment whereby the availability of artificial perches is manipulated to test this hypothesis.

An alternative explanation for the positive effect of habitat visibility on defense efficiency could be that predation risk may be higher in high-visibility environments and that few individuals are willing to defend food sources or challenge defenders under such a risk. Hamilton and Dill (2002) tested this hypothesis in Zebrafish (*Danio rerio*) and found that food monopolization increased in habitats perceived as riskier compared to safer habitats of similar visibility. However, males in our study area often used fully exposed perches and chased competitors high in the air and far from cover. Moreover, males made a disproportionate use of feeders in open habitats (Table 2), suggesting that predation avoidance, if high-visibility environments are associated with high predation risk, is not the primary determinant of settlement decisions by males. Still, even if hummingbirds are presumed to experience low predation (Robinson et al. 1996), susceptibility to predation should not be equated with a lack of sensitivity to this risk (Lima 1991).

### Influence of natural food sources

As nectar sucrose concentration was maintained constant, at least in the first 2 years of the study, differential use of feeders was ultimately determined by surrounding habitat structure. One variable not necessarily related to habitat structure that could have influenced feeder use is the availability of natural food sources. Negative correlations between flower abundance and feeder use have been observed in other hummingbird species (Inouye et al. 1991; McCaffrey and Wethington 2008). Yet, the only plant species known to be important for ruby-throated hummingbirds and which occurred within our study site, namely *Impatiens capensis* (Bertin 1982), was in low abundance and present only for a short period of time at the end of the season. In addition to rarely observing pollen on the bill and forehead of individuals, except at the end of the season, we never observed ruby-throated hummingbirds feeding from sap-filled holes in trees maintained by yellow-bellied sapsuckers (*Sphyrapicus varius*; Southwick and Southwick 1980) and rarely observed them feeding from natural flowers within our study site. Insects were often caught in flight, but we believe that natural nectar consumption within our study site was negligible compared to the use of our supplied nectar.

### Measuring resource defense and monopolization in nature

Individuals are often constrained to feed from a single food patch in spatially confined, experimental laboratory setups to facilitate the measurement of resource defense and monopolization (e.g., Chapman and Kramer 1996; Basquill and Grant 1998). In nature, however, competition among individuals of varying quality and taking place within heterogeneous landscapes likely results in some individuals being forced to less favorable habitats where competitive pressure is reduced. Resource monopolization in these poorer habitats can thus appear high, but may be due to low competitive pressure rather than to strong resource defense. Therefore, the degree of use of the different habitats needs to be considered when quantifying resource monopolization in natural environments. By tracking visits to feeders through RFID, we were able to circumvent the problems associated with variable habitat use by taking into account the relative spatial concentration of individuals at feeders. This measure allowed us to quantify the baseline level of visits by competitors at feeders (i.e., when a focal individual's spatial concentration ∼ 0) and thereby assess the reduction in the number of visits made by competitors caused by the focal individual, which allows to differentiate between feeder defense and monopolization.

Our study shows the importance of studying territorial and resource defense behaviors within a multivariate context as many variables will influence the level to which animals can monopolize space or food resources. Moreover, our study highlights the importance of considering the space use of individuals as it strongly affected feeder monopolization by male ruby-throated hummingbirds in our system. Territoriality is often viewed as a static behavior whereby individuals rarely leave their territories. However, studies show that territorial animals often have a more complex space use than what is assumed by such a rigid view of territoriality and that factors other than food resource distribution can affect this use (e.g., Sikkel and Kramer 2006; Lenda et al. 2012).Thus, following individuals in space, which is often neglected because of the difficulty of tracking movements over large spatial and temporal scales, appears crucial to gain a better understanding of why and how territorial animals can monopolize resources in the wild and thereby to provide greater insights into the costs and benefits of different spatial strategies, which ultimately impact fitness and population dynamics (Sutherland 1996).

## Figures and Tables

**Figure A1:**
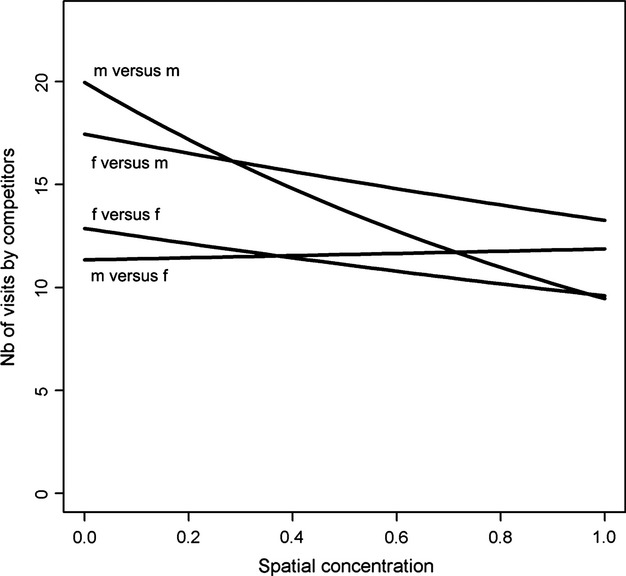
Predicted number of visits by competitors (NVC) against the spatial concentration of focal Ruby-throated Hummingbirds for the four sex combinations studied in Cleveland County, Quebec (Canada), 2007–2009. Predictions are derived from model-averaged, linear mixed models with feeder ID and the intercept and slope of spatial concentration for focal individual ID as random effects. Other numeric variables were fixed to their mean value, and factors were fixed to their reference level.

**Figure A2:**
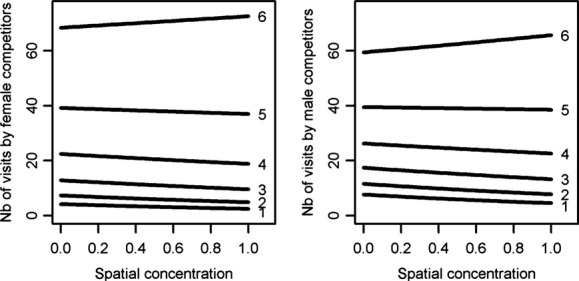
Model-averaged predictions for the number of visits made by female or male ruby-throated hummingbird competitors (NVC) in Cleveland County, Quebec (Canada), 2007–2009, in relation to the spatial concentration of focal female and the number of female or male competitors. Predictions are derived from linear mixed models with feeder ID and the intercept and slope of spatial concentration for focal individual ID as random effects. The response variable was log-transformed. Other numeric variables were fixed to their mean value, and factors were fixed to their reference level.

**Figure B1:**
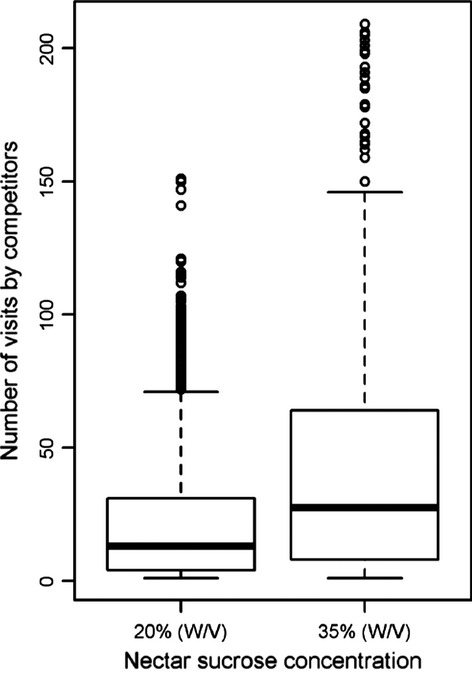
Number of visits made by adult male Ruby-throated Hummingbird competitors at feeders potentially defended by males when containing nectar with a low (20% W/V) or a high (35% W/V) sucrose concentration in Cleveland County, Quebec (Canada). Data shown are restricted to the period when sucrose concentration was manipulated (i.e., between 10 June 2009 and 11 August 2009).

## APPENDIX

### Appendix A

Model selection and effect of explanatory variables on the number of visits by Ruby-throated Hummingbird competitors (NVC) for sex combinations other than male vs. males (see Table2).

**Table A1:** Model selection and explanatory variables composing the 10 models put in competition by AICc for modeling the number of visits by ruby-throated hummingbird competitors in Cleveland County, Quebec (Canada), 2007–2009. Variables included and omitted from a model are indicated by a cross and a circle, respectively. The same set of models was used for the three sex combinations. Feeder rank is based on the sex of competitors. Akaike weights (*w*_i_) represent the probability that a particular model best describes the data. The response variable was log-transformed and modeled with linear mixed-effect models with feeder ID and the intercept and slope of spatial concentration for focal individual ID as random effects.

Variables	1	2	3	4	5	6	7	8	9	10
Year	x	x	x	x	x	x	x	x	x	x
Grid usage	x	x	x	x	x	x	x	x	x	x
nb of competitors	x	x	x	x	x	x	x	x	x	x
Feeder rank	x	x	x	x	x	x	x	x	x	x
Nectar sucrose concentration	x	x	x	x	x	x	x	x	x	x
Spatial concentration	o	o	x	x	x	x	x	x	x	x
Daily nb of visits	o	o	x	o	x	x	x	x	x	x
Spatial stability	o	o	x	x	o	x	x	x	x	x
Openness	o	x	x	x	x	x	o	x	x	x
Lateral visibility	o	x	x	x	x	x	o	x	x	x
Spatial concentration:daily nb of visits	o	o	o	o	x	x	x	x	x	x
Spatial concentration:nb of competitors	o	o	o	x	x	o	x	x	x	x
Spatial concentration:spatial stability	o	o	o	x	o	x	x	x	x	x
Spatial concentration:openness:lateral visibility	o	o	o	x	x	x	o	o	x	x
Spatial concentration:feeder concentration	o	o	o	o	o	o	o	o	o	x
Males versus females competitors										
ΔAICc	53.96	50.92	5.44	13.37	1.15	2.34	3.07	0.00	3.31	5.13
*w*_*i*_	0.00	0.00	0.03	0.00	0.23	0.13	0.09	0.41	0.08	0.03
Females versus females competitors										
ΔAICc	263.46	253.05	9.00	0.80	6.71	5.00	8.18	0.44	0.75	0.00
*w*_*i*_	0.00	0.00	0.00	0.20	0.01	0.03	0.01	0.24	0.21	0.30
Females versus males competitors										
ΔAICc	45.36	41.08	11.34	9.66	3.92	6.86	3.69	0.00	3.20	4.85
*w*_*i*_	0.00	0.00	0.00	0.01	0.09	0.02	0.10	0.61	0.12	0.05

**Table A2:** Model-averaged coefficients (coef), unconditional standard errors (SE) and 95% confidence intervals (lower CI and upper CI) for the explanatory variables used for modeling the number of visits made by adult female Ruby-throated Hummingbirds competitors (NVC) at feeders potentially defended by a female in Cleveland County, Quebec (Canada), 2007–2009. The response variable was log-transformed and modeled using linear mixed models with feeder ID and the intercept and slope of spatial concentration for focal individual ID as random effects. Results are based on 75 focal individuals monitored for a total of 1709 bird-days and 5886 bird-feeder-days.

Variables	Coef	SE	Lower CI	Upper CI
Year 2008	−0.21603	0.04807	−0.31025	−0.12181
Year 2009	−0.47663	0.07031	−0.61445	−0.33882
Grid usage	0.00714	0.00147	0.00426	0.01003
nb of competitors	0.55635	0.01597	0.52504	0.58766
Feeder rank for females	−0.00821	0.00267	−0.01344	−0.00297
Nectar sucrose concentration (high)	0.76434	0.11555	0.53785	0.99082
Spatial concentration	−0.23988	0.33744	−0.90126	0.42150
Daily nb of visits	0.00189	0.00094	0.00005	0.00372
Spatial stability	−0.37015	0.16959	−0.70254	−0.03775
Openness (open)	−0.55542	0.17047	−0.88954	−0.22130
Lateral visibility	0.00004	0.00355	−0.00693	0.00700
Spatial concentration:daily nb of visits	−0.00368	0.00247	−0.00852	0.00115
nb of competitors:spatial concentration	0.11757	0.04747	0.02453	0.21062
Spatial concentration:spatial stability	−0.47474	0.50005	−1.45485	0.50536
Spatial concentration:feeder concentration (high)	−0.44004	0.26707	−0.96350	0.08342
Spatial concentration:openness (close):lateral visibility	0.02319	0.01212	−0.00056	0.04694
Spatial concentration:openness (open):lateral visibility	0.00308	0.00343	−0.00364	0.00980

**Table A3:** Model-averaged coefficients (coef), unconditional standard errors (SE) and 95% confidence intervals (lower CI and upper CI) for the explanatory variables used for modeling the number of visits made by adult female Ruby-throated Hummingbirds competitors (NVC) at feeders potentially defended by a male in Cleveland County, Quebec (Canada), 2007–2009. The response variable was log-transformed and modeled using linear mixed models with feeder ID and the intercept and slope of spatial concentration for focal individual ID as random effects. Results are based on 86 focal individuals monitored for a total of 2497 bird-days and 7329 bird-feeder-days.

Variables	Coef	SE	Lower CI	Upper CI
Year 2008	−0.01387	0.04091	−0.09406	0.06632
Year 2009	−0.10482	0.05091	−0.20461	−0.00503
Grid usage	0.00930	0.00114	0.00706	0.01153
nb of competitors	0.64941	0.01322	0.62350	0.67531
Feeder rank for females	−0.00266	0.00191	−0.00640	0.00108
Nectar sucrose concentration (high)	0.31911	0.06423	0.19323	0.44500
Spatial concentration	0.03194	0.20508	−0.37002	0.43389
Daily nb of visits	−0.00108	0.00060	−0.00225	0.00010
Spatial stability	0.13970	0.11458	−0.08488	0.36428
Openness (open)	−0.42793	0.17317	−0.76734	−0.08851
Lateral visibility	−0.00044	0.00350	−0.00729	0.00641
Spatial concentration:daily nb of visits	−0.00265	0.00144	−0.00547	0.00017
nb of competitors:spatial concentration	0.04258	0.04048	−0.03677	0.12193
Spatial concentration:spatial stability	0.02904	0.31582	−0.58998	0.64805
Spatial concentration:feeder concentration (high)	−0.10513	0.24035	−0.57621	0.36595
Spatial concentration:openness (close):lateral visibility	0.01095	0.01329	−0.01509	0.03699
Spatial concentration:openness (open):lateral visibility	0.00001	0.00192	−0.00375	0.00377

**Table A4:** Model-averaged coefficients (coef), unconditional standard errors (SE) and 95% confidence intervals (lower CI and upper CI) for the explanatory variables used for modeling the number of visits made by adult male Ruby-throated Hummingbirds competitors (NVC) at feeders potentially defended by a female in Cleveland County, Quebec (Canada), 2007–2009. The response variable was log-transformed and modeled using linear mixed models with feeder ID and the intercept and slope of spatial concentration for focal individual ID as random effects. Results are based on 75 focal individuals monitored for a total of 1539 bird-days and 4924 bird-feeder-days.

Variables	Coef	SE	Lower CI	Upper CI
Year 2008	−0.17667	0.05680	−0.28800	−0.06535
Year 2009	−0.49535	0.08018	−0.65251	−0.33818
Grid usage	0.01299	0.00186	0.00935	0.01664
nb of competitors	0.40796	0.01434	0.37985	0.43607
Feeder rank for males	−0.00259	0.00519	−0.01276	0.00757
Nectar sucrose concentration (high)	−0.02277	0.12597	−0.26966	0.22413
Spatial concentration	−0.75438	0.38194	−1.50298	−0.00578
Daily nb of visits	0.00021	0.00117	−0.00208	0.00250
Spatial stability	−0.35625	0.19069	−0.73001	0.01751
Openness (open)	0.46342	0.19119	0.08869	0.83816
Lateral visibility	−0.00605	0.00364	−0.01318	0.00107
Spatial concentration:daily nb of visits	−0.00921	0.00325	−0.01557	−0.00285
nb of competitors:spatial concentration	0.12489	0.04944	0.02799	0.22179
Spatial concentration:spatial stability	0.96033	0.56992	−0.15672	2.07737
Spatial concentration:feeder concentration (high)	0.28712	0.45861	−0.61176	1.18600
Spatial concentration:openness (close):lateral visibility	−0.01200	0.01546	−0.04231	0.01831
Spatial concentration:openness (open):lateral visibility	−0.00158	0.00373	−0.00889	0.00573
